# Data-Driven Insights into E-Learning: A Comprehensive Review of Eye-Tracking Applications in Learning Systems

**DOI:** 10.3390/jemr19020041

**Published:** 2026-04-17

**Authors:** Safia Bendjebar, Yacine Lafifi, Rochdi Boudjehem, Aissa Laouissi

**Affiliations:** 1 LabSTIC Laboratory, University of 8 May 1945 Guelma, P.O. Box 401, Guelma 24000, Algeria; lafifi.yacine@univ-guelma.dz (Y.L.); boudjehem.rochdi@univ-guelma.dz (R.B.); 2 Department of Mechanical Engineering, Faculty of Science and Technology, University of Mohamed El Bachir El Ibrahimi, Bordj Bou Arreridj 34000, Algeria

**Keywords:** eye tracking, e-learning, cognitive processes, visual attention, learning styles, cognitive load, engagement

## Abstract

In the last few years, universities have increasingly implemented online learning environments, allowing students to study at their own pace. These environments utilize technological tools and implement methods to support training, deliver content, and promote the acquisition of new knowledge and skills. As an example of these technologies, eye tracking has emerged as a powerful tool for studying visual attention, cognitive processes, and learning behaviors. The main aim of this study is to provide a scoping review of recent eye-tracking research across diverse learner populations, ranging from K-12 students to university-level learners and educators. The present study examined recent advances in eye-tracking technologies, focusing on their potential, especially when combined with artificial intelligence (AI) techniques such as machine learning. It analyzed 54 empirical studies in the last few years, highlighting their applicability, strengths, and limitations. The research findings highlight the promise of eye-tracking technology to transform educational practices by providing data-driven insights regarding student behavior and cognitive processes. Future research must address implementation and data-analysis challenges to maximize the educational benefits of eye tracking.

## 1. Introduction

Eye tracking is a technology that monitors the location and duration of a person’s gaze, providing valuable insights into visual attention and cognitive processes across psychology, education, and human-computer interaction [1]. The application of eye tracking extends beyond e-learning, finding utility in various domains. For instance, in marketing, this technology helps understand consumer behavior and preferences by analyzing their visual attention to advertisements. In healthcare, it assists in diagnosing neurological conditions and assessing cognitive function. In usability testing, it aids in identifying areas of difficulty and improving the design of user interfaces. Integrating this technology into e-learning systems has significant potential to improve the learning experience. Teachers can understand learners’ interactions with digital materials by analyzing gaze patterns. This data can personalize learning experiences afterwards, optimize instructional strategies, and create more effective and engaging e-learning environments. Beyond external visual stimuli, eye movements are deeply intertwined with internal cognitive processes, such as memory retrieval and mental imagery, reflecting the underlying neural network activity during a complex task execution [2].

This technology captures key ocular metrics, including fixations, saccades, and gaze points, to analyze user behavior and attention. Collecting data on eye movements provides researchers with insights into visual attention, engagement, and cognitive processes [3]. These features are often extracted using various tools and software solutions. For instance, specialized software can provide valuable insights into user behavior and identify users who focus their attention on a webpage or document, revealing areas of high interest and potential distractions [4]. Additionally, some studies have explored the use of webcam-based eye-tracking systems to analyze students’ attention during learning activities, demonstrating their potential for scalable, cost-effective educational applications [5]. Beyond traditional screens, this technology is increasingly integrated into immersive environments, such as Virtual Reality (VR), to monitor and improve student engagement. For instance, eye-gaze data can be used to detect levels of distraction due to factors such as stress or mind-wandering [6]. Recent findings suggest that these systems can effectively replicate real-world eye-dominance behaviors in VR, providing a consistent framework for behavioral analysis across varying viewing distances [7].

Furthermore, eye tracking has significantly enhanced classroom management. Training teachers to effectively use eye contact can positively influence their ability to foster a structured and engaging classroom environment [8]. Eye-tracking enables the assessment and refinement of classroom management skills by analyzing instructors’ gaze patterns, including where they direct their attention, how frequently they engage with students through eye contact, and how their visual focus shifts between students, teaching materials, and classroom technology [9]. Moreover, integrating eye-tracking data with additional behavioral metrics, such as gesture and speech analysis, can provide a comprehensive understanding of teaching dynamics, thereby aiding the development of personalized feedback for educators to enhance their classroom management skills [3]. This integration is facilitated by machine learning techniques that process and analyze complex datasets from multiple sources to improve the accuracy of emotion and cognitive state recognition [10]. Machine learning and Internet of Things (IoT) technologies significantly improve eye-tracking applications by enabling more accurate detection of gaze positions and motions and reducing manual recalibration [11]. These technologies can help create more personalized, engaging, and compelling learning experiences.

This scoping review identifies the most effective eye-movement metrics for characterizing and understanding visual attention. It defines eye-tracking systems using widely accepted sources, ensuring a clear understanding of their capabilities and limitations, and guides our critical evaluation of the existing literature on eye-movement metrics and their relationship to the underlying processes of visual attention.

This study aims to provide a comprehensive overview of recent advances in eye-tracking applications in e-learning environments, focusing on research from the last five years. The scope of this review is framed by the following research questions: (1) How does eye-tracking technology enhance personalized learning experiences in e-learning environments? (2) What limitations exist in current eye-tracking applications within educational settings? (3) Can eye-tracking data be effectively integrated with other data (e.g., learning management system data, student performance data, etc.) to provide a more comprehensive understanding of the learner experience in e-learning environments?

## 2. Related Work

In recent years, several review studies have examined the integration of eye-tracking technology in educational research. However, most of these studies focus on the technological evolution of sensors or on broad pedagogical outcomes, leaving a gap in the methodologies used for data-driven learning analytics. Table 1 provides a comparison between previous review studies and the present study.

**Figure 1:**
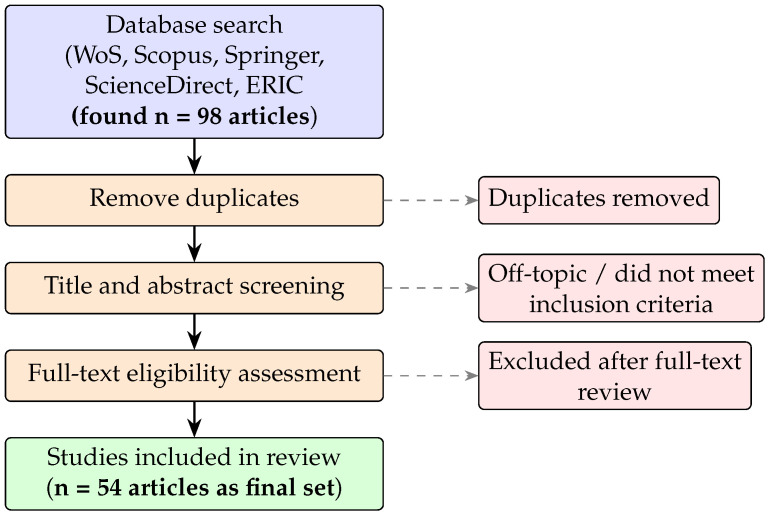
Systematic review process following PRISMA guidelines.

In this scoping review, we systematically analyze the existing literature on eye-tracking applications in e-learning environments, categorizing studies based on their global objectives (e.g., learner behavior analysis, learning content optimization, behavioral assessment). We also evaluate the methodologies used for real-time data processing and automated feedback loops, particularly in webcam-based and VR learning environments. Additionally, we address the need for learner-adaptive analytics by examining how ocular metrics are interpreted differently based on learner levels and expertise (see Figure 1).

As shown in Table 1, previous reviews primarily focus on specific theoretical aspects such as cognitive load, multimedia learning principles, and mathematical cognition. While these studies provide essential foundational knowledge, they provide works that lack actionable methodological guidance for modern, decentralized learning environments. Our work distinguishes itself from these prior reviews in three fundamental ways:

- Categorization by Global Objectives: Unlike the mapping approach of ref. [18], or the subject-specific focus of [14], we categorize the literature according to the Global Objective within the e-learning (e.g., learner behavior, learning content, or behavioral assessment). This provides a functional roadmap for developers and researchers to align eye-tracking integration with specific pedagogical goals.
- Methodological Real-Time Implementation: While ref. [15] identifies the lack of real-time interaction in remote learning as a critical gap, our review systematically analyzes the automated feedback loops and data processing pipelines required, specifically within webcam-based and VR environments.
- Diverse Population of Learners: Moving beyond the simple user assumption common in studies like [12], we specifically address how ocular metrics must be interpreted differently based on learner levels. This focus on learner-adaptive analytics is essential for creating truly personalized and intelligent tutoring systems.
- Our review does not simply compare articles. It provides a practical guide to the tools (Table 2) for moving from theory to real-world implementation.

Furthermore, our study highlights the role of automated analytics and standardized study selection procedures to support real-time learning analytics.

## 3. Available Eye-Tracker Tools

Eye-tracking technology has revolutionized the study of human behavior, particularly in fields like user experience, cognitive psychology, and educational research. Various eye-tracking devices are available, ranging from screen-based systems, such as the SMI RED250 and Tobii TX300, to wearable devices, such as the Tobii Pro Glasses, and budget-friendly options, such as the Eye Tribe.

Table 2 compares different commercial and open-source eye-tracking tools.

## 4. Eye-Tracking Measurement Metrics

Eye movements primarily consist of fixations and saccades. During fixations, the eyes remain relatively stationary, allowing for the acquisition and processing of visual information. A fixation is typically defined as a period of at least 100 ms where eye movement remains below a specific threshold (e.g., 1°). However, the reliability of this classification depends on the system’s spatial resolution, and the hardware’s precision must be significantly finer than the chosen threshold to effectively distinguish fixations from noise or small movements. Consequently, systems with low spatial accuracy (e.g., 1°, such as some webcam-based trackers) may not be suitable for reliable fixation assessment using standard criteria [27].

We can extract additional features from this metric, such as average fixation duration, time to first fixation, total fixation duration, etc. [12,28]. Note that the precision of these temporal metrics depends on the sampling rate of the eye-tracking system (e.g., a tracker operating at 60 Hz provides a resolution of 16.7 ms, which defines the minimum detectable change in fixation duration). In e-learning, these metrics provide valuable insights into how learners allocate visual attention during learning tasks. Long fixation durations typically indicate a higher level of reasoning with the observed content [29].

A saccade is a rapid, involuntary eye movement that shifts gaze between fixations, periods of relatively stable gaze. This rapid movement enables efficient processing of visual information by quickly shifting focus from one point to another. Based on this metric, we compute relevant features that include saccade rate, average saccade length, average saccade amplitude, and average saccade velocity [30].

In fact, the saccade velocity requires adequate temporal and spatial resolution. Because saccades are brief events (typically 20–50 ms), accurate velocity estimation generally requires high sampling rates (e.g., more than 250 Hz) and sub-degree spatial accuracy. Eye-tracking systems operating at lower sampling rates (e.g., 60 Hz) may provide coarse estimates of saccade amplitude or rate but are often insufficient for precise measurements of peak velocity. In the e-learning context, velocity is primarily used as an indicator of cognitive fatigue or arousal, as a decline in velocity often correlates with decreased learner engagement. It can provide complementary information about visual search strategies, reading patterns, and cognitive load [31].

Researchers often employ heatmaps to analyze gaze fixation data. This visualization assigns colors to different regions of the visual field constructed from the density of fixations. Warmer colors, such as red and orange, indicate higher fixation densities, whereas cooler colors, such as blue and green, indicate lower densities. The heatmap peak, typically shown in red, indicates the area that received the most attention. Unlike scan paths, heatmaps do not provide information about the sequence of fixations [5]. The following Table 3 provides a comprehensive list and description of the most commonly used metrics.

## 5. Eye-Tracking Methodologies in E-Learning

**Figure 2:**
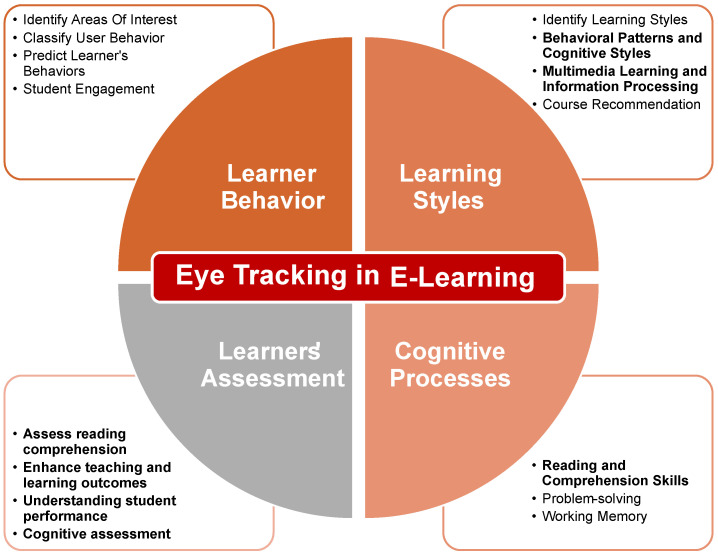
Eye-tracking methodologies in e-learning systems.

This study conducted a scoping review to synthesize research on the application of eye-tracking methods in e-learning, drawing on the most recent publications. The focus was on studies that specifically examined the use of eye-tracking technology within eye-tracking applications for (see Figure 2):

- Modeling learner’s behavior,
- Cognitive processes,
- Learning styles,
- The learning content,
- Learners’ assessment.

This study conducted a scoping review, searching prominent databases, including Web of Science, Springer, Scopus, ScienceDirect, and Education Sources, covering the period from 2020 to 2026.

The search strategy employed specific Boolean strings, such as (‘eye-tracking’ OR ‘gaze-based’ OR ‘eye-movement’) AND (‘e-learning’ OR ‘online learning’ OR ‘educational technology’) AND (‘metrics’ OR ‘behavior’ OR ‘measument’).

The inclusion criteria were peer-reviewed journal articles that specifically investigated the application of eye-tracking technology within specialized e-learning systems. To ensure the reliability of the findings, a structured data extraction schema was used. The authors independently reviewed the selected papers to identify the metrics and objectives, and then conducted peer-validation sessions to resolve any discrepancies in categorization. The final selection is presented chronologically and thematically to illustrate the progression and trends in the field (see Appendix A, Table A1 for including the rationale).

### 5.1. Eye-Tracking Applications for Modeling Learner Behaviors

Analyzing learner behavior examines and interprets learner data generated within a learning environment to inform instructional improvement [32]. In the context of eye-tracking research, learner behavior refers to visual interactions with instructional materials. In this study, we analyze studies using the metrics defined in Table 2. For example, increases in average fixation duration or decreases in saccade rate can signal student engagement in e-learning systems. These insights inform instructional improvement by identifying Areas of Interest (AOIs) that cause learner confusion, thereby enabling better alignment with learners’ cognitive capacity. Such analysis also allows timely pedagogical interventions when disengagement or cognitive overload is detected. Table 4 presents a structured overview of studies that applied eye tracking in e-learning, categorized under behavior process.

#### 5.1.1. Identify Areas of Interest

Researchers have widely applied eye-tracking technology to identify Areas Of Interest (AOI) by tracking where users fixate. It can pinpoint the most engaging elements of a course. The ability to identify and analyze areas of interest in e-learning environments using eye tracking provides insights into how learners engage with content [20].

In another context, Wyss et al. [33] analyzed eye-tracking data alongside post-hoc think-aloud verbalizations to examine teachers’ professional vision for noticing and interpreting significant classroom interactions. In their study, participants viewed a video of a lesson containing a critical incident (a pivotal event impacting the teaching-learning flow). The findings revealed that experienced teachers exhibited stronger professional vision, and they detected the critical incident more quickly (shorter time to first fixation). They made more frequent fixations on the critical incident than novices. Chen et al. [34] employed a commercial eye-tracking tool to collect and analyze students’ eye-movement data during Facebook Live streaming, identifying participants’ areas of interest and behavior, providing insights into visual attention and cognitive engagement. This work can guide the design of more effective live-streamed educational content to maximize attention, behavior, and interaction.

#### 5.1.2. Classify User Behavior

Classifying learner behaviors based on eye-tracking data provides educators with insights into information processing, engagement, and learner–environment interactions, thereby enabling the development of highly personalized and adaptive learning systems [35]. These classifications facilitate the identification of distinct behavioral profiles. In this work [35], for example, the Explorer (Global) profile is characterized by long saccades and short fixation durations, reflecting rapid scanning of the content to grasp its overall structure.

GazeReader, a system developed by Ding et al. [36], is designed to track and analyze learners’ behavior during reading tasks of English as a Second Language (ESL), specifically focusing on how learners interact with unknown words. The authors incorporate term frequency, part-of-speech, and named entity recognition to improve the accuracy of unknown-word detection. Analyzing learners’ gaze patterns, the machine learning model in this work can predict the specific word that the learner is struggling with.

A recent study explored the potential of scalable eye-tracking technology to support neurodivergent students in educational settings. This research, conducted by [37], evaluated the effectiveness of webcam-based eye tracking in measuring differences in reading and online thought patterns. The findings demonstrated that webcam-based eye-tracking could provide reliable information about students’ reading behaviors, including distinguishing between students who have and have not read the text previously, even in noisy classroom environments.

#### 5.1.3. Predict Learners’ Behaviors

Eye-tracking technology helps understand learners’ attention and behavior in multimedia contexts and can be used for early intervention [28,38]. As our lives become increasingly digital, AI (Artificial Intelligence) can tap into the vast amounts of data generated online to anticipate consumer trends and preferences [39].

Chettaoui et al. [40] integrated eye-tracking data with learners’ profiles, academic performance, and time-on-task data to predict learners’ learning outcomes in an embodied learning activity. This research demonstrates the significant potential of eye tracking to enhance our understanding of student behavior and engagement, thereby informing the development of more effective and personalized assessments in embodied learning contexts.

A recent study by Kok et al. [41] examined three prerequisites for using gaze visualizations to inform teachers about students’ comprehension during video lectures. These prerequisites included (1) establishing a correlation between gaze measures and student performance on subsequent assessments, (2) investigating the capacity of laypeople to predict student performance on post-tests based on the presented gaze visualizations, and (3) evaluating the impact of various gaze visualization techniques on the accuracy of these predictions.

The integration of Artificial Intelligence (AI) with eye tracking represents a significant trend in the state of the art. For instance, Šola et al. [42] used AI to process gaze recordings from a large-scale dataset, achieving high accuracy (97–99%) in predicting students’ preferences and cognitive demand from visual interactions with digital layouts. This high degree of precision highlights the potential of Big Data and pre-trained neuromarketing models in predicting user attention.

#### 5.1.4. Improving Student Engagement

Student engagement in e-learning refers to the degree to which a learner is actively involved in the learning process, exhibiting attention, curiosity, and participation in digital learning activities [43]. Eye tracking offers a unique method for objectively measuring student engagement in e-learning environments. Analyzing eye-movement metrics such as fixations and saccades, researchers can monitor students’ attention and engagement during online and remote learning sessions, providing valuable data to improve teaching strategies and to support early intervention for attention-related issues [44,45].

To measure student engagement and attention levels to enhance online learning, Hossen and Uddin [46] developed a system that incorporated advanced features, including facial recognition, hand tracking, and mobile phone detection, to analyze student behavior in real time. This study employed the extreme gradient boosting (XGBoost) algorithm to detect and predict attention levels, achieving an accuracy of 99.75%. A key factor behind this result is the limited sample size of 30 participants. While the dataset contains 4000 records, they originate from a small, homogeneous group of students, which significantly reduces the variance in behavior that the model must classify.

Furthermore, Slim and Hartsuiker [47] have employed customized eye-tracking systems, including the PCIbex software developed by Zehr and Schwarz in 2018 [48], with the WebGazer algorithm proposed in 2016 by Papoutsaki and his team to tailor their studies to specific requirements. WebGazer is a JavaScript library using devices’ built-in webcams to track user eye movements. It does not require any specific hardware or a dedicated eye-tracking device. This library has been widely used in platforms that require eye-tracking functionality.

Qi et al. [49] developed a novel tool that leverages a cascaded analysis network to address the challenge of maintaining student engagement in online learning environments. The model effectively assesses student attention and engagement levels by analyzing gaze behavior, facial expressions, and physical actions. This research has the potential to significantly improve the efficiency of online learning by providing real-time alerts that can help educators identify and address student distractions.

**Table 4:** Analysis of eye-tracking studies in e-learning according to behavior processes.

Type of Affect	Author(s)	Objective(s)	Task(s)	Explored Feature(s)	Eye-Tracking Model	Participants
Identifying areas of interest	[33]	Analyze teachers’ professional visions and compare those of novice and experienced teachers.	Watching a video depicting a critical incident in a school lesson.	Fixation count; fixation duration	GazePoint Analysis Software Prof (Software)	28
	[34]	Understand how students engage with live e-commerce content on Facebook Live.	Observing content presented on a live platform.	Initial fixation latency; initial fixation duration; fixation duration; total number of fixations	Eyeglass tool (Wearable)	31
	[50]	Support teachers’ professional vision.	Participate in a classroom simulation while wearing a mobile eye tracker.	Average fixation duration	Tobii Pro Glasses 3 (Wearable)	4
	[51]	Investigate how college students allocate visual attention during online live teaching.	Watching a presentation during an online live teaching session.	Fixation duration; percentage of total fixation time	–	78
Classify user behavior	[36]	Recognize English words unknown to learners.	Read texts with unknown words while a webcam tracked their gaze.	Fixation duration; number of regressions	Webcam-based eye-tracking (Webcam)	12
	[37]	Monitor thought processes and reading behaviors of neurodivergent learners.	Read a text and participate in a reflection exercise.	Fixations; saccades; heatmaps	Webcam-based eye-tracking (Webcam)	43
	[52]	Classify user behaviors on websites.	Web reading or searching for web elements.	Fixations; saccades	Tobii Studio Software (Software)	30
Predict behaviors	[40]	Predict learning performance during a tangible learning activity.	Participate in an embodied learning activity on human body anatomy.	Fixation count; fixation duration	Web camera (Webcam)	110
	[41]	Inform teachers about students’ engagement and comprehension during online lectures.	Watch an instruction video on electrocardiogram interpretation.	Average proportion of fixation; maximum gaze coupling	SMI RED Mobile eye tracker (Remote)	36
	[35]	Enhance the design of college magazines and improve user experience.	Interact with online and PDF versions of magazines.	Fixations; heatmaps	Tobii X2 (Remote)	AI dataset (180,000 samples)
Student Engagement	[53]	Capture participants’ attention and engagement.	Watch videos and answer a questionnaire.	Fixations; saccades; heatmaps; scanpaths	Web camera (Webcam)	40
	[46]	Measure and analyze student behavior in virtual learning environments.	Participate in online classes as usual.	/	Web camera (Webcam)	30
	[47]	Investigate the impact of instructional strategies on student engagement.	Watch a video lecture.	Fixation duration; saccade rate; pupil dilation	PCIbex and WebGazer (Webcam)	57
	[49]	Measure and analyze student behavior in virtual learning environments.	Complete online learning activities.	Gaze angles	Camera (Webcam)	50

### 5.2. Eye-Tracking Applications for Cognitive Processes

To improve the e-learning process, it is crucial to understand how the mind works. Cognitive processes like attention, perception, memory, and problem-solving play a key role in effective learning [54]. Integrating eye-tracking into e-learning systems enables insights into how learners encode and retain information.

#### 5.2.1. Improving Reading and Comprehension Skills

Reading and comprehension are fundamental for academic and personal growth [55]. Strong reading and comprehension skills are crucial for success in academia and professional settings, enabling individuals to extract meaning from text and apply their knowledge effectively. Eye-tracking technology offers real-time insights into reading behavior and comprehension by analyzing eye movements and gaze patterns [17]. Integrating gaze data and physiological responses, Santhosh et al. [56] successfully measured and predicted user interest levels while reading newspapers. To do so, they evaluated the performance of different machine learning models (CNN-LSTM, manual feature extraction) for interest-level prediction. Thirteen university students participated in an experiment where they read 18 newspaper articles. Gaze data were collected using an SMI eye-tracker, and physiological data were concurrently recorded.

Hutt et al. [5] developed a scalable solution for monitoring cognitive states during learning. They used a webcam-based eye-tracker (Webgazer) to identify task-unrelated thoughts and comprehension during online reading tasks. Their experimentation involved analyzing performance under various conditions to investigate how factors like lighting, glasses, and dataset diversity affect the accuracy and generalizability of the results.

To create more personalized and effective e-learning experiences, Bostan and Ozcelik [57] demonstrated the impact of pre-questions on learning, showing that asking integrative questions before studying can enhance learning outcomes. Analyzing eye movements using eye-tracking technology, researchers gained insights into the cognitive processes underlying the learning benefits of pre-questioning, suggesting their value as an active learning strategy.

#### 5.2.2. Problem-Solving Using Eye-Tracking Technology

Problem-solving is a fundamental skill that permeates all levels of education. Da Silva Soares et al. [16] comprehensively review eye-tracking applications. This study aims to elucidate the potential of this non-invasive, real-time measurement method to improve mathematics instruction and learning. In mathematics education research, eye-tracking offers significant benefits. It allows researchers to study cognitive processes, uncover underlying mental representations, and assess subconscious aspects of mathematical thinking [14]. In this context, Türkoğlu and Yalçınalp [58] analyzed how university students solve geometry problems using eye-tracking technology. The research findings emphasized the crucial role of understanding and planning in problem-solving situations and revealed that those who converged were more effective and invested in these processes.

A recent study highlighted the transformative potential of visualization tools in enhancing targeted educational interventions. This research, conducted by Wei et al. [59], introduced a novel system that integrates the visualization of elementary school students’ academic performance with detailed eye movement data. This innovative approach enables researchers to gain deeper insights into students’ attention patterns, cognitive engagement, and problem-solving strategies during learning activities. Identifying these critical behavioral and cognitive trends, the system supports designing more personalized and adaptive learning support.

In Educational Neuroscience, Da Silva Soares et al. [60] demonstrated that teachers can accurately predict students’ success using eye-tracking data, underscoring the potential of this technology to provide valuable insights into students’ problem-solving approaches. Teachers can better understand how students interact with learning materials, identify potential areas of difficulty, and tailor their instruction to individual student needs. These findings have significant implications for personalized learning, as they suggest that eye-tracking data can inform teaching strategies and improve student outcomes.

#### 5.2.3. Studying Working Memory

Eye-tracking technology has become a powerful tool for studying working memory by analyzing gaze behavior and visual attention. Measuring these patterns, researchers gain valuable insights into the development of human working memory and its role in determining subjective cognitive load. Abeysinghe [61] further solidified this understanding by demonstrating a strong connection between human eye movement patterns and working memory processes. To investigate the potential of eye tracking as a remediation strategy for children with ASD, ADHD, and specific learning disorders. Chan et al. [62] conducted a study on this problem. They assessed the impact of eye-tracking training on cognitive functions, particularly learning and memory, by evaluating student performance on the Hong Kong List Learning Test. In her 2024 dissertation, Bühler [63] developed an automated methodology for assessing attention-related processes using a multimodal approach that integrates eye-tracking, computer vision, and machine learning techniques. Addressing critical challenges, including fine-grained assessment, data quality, and generalizability in real-world educational contexts, this work contributes novel approaches to understanding and improving attention-related processes in learning environments at scale. These advancements have significant implications for enhancing instructional design and personalizing learning experiences. Table 5 summarizes research studies that have utilized eye-tracking technology to investigate various aspects of cognitive processes.

### 5.3. Eye-Tracking Application for Learning Styles

Over the years, learning styles have been defined as the ways individuals prefer to learn and process information. Recognizing and accommodating these diverse styles is crucial in effective e-learning design [67,68].

A brief review of relevant studies on learning styles was published in 2020 by Wibirama et al. [13], which reviewed their strengths and limitations and provided insights into potential methods for detecting learning styles in multimedia learning. They prove that eye-tracking with machine learning is a valuable tool for enhancing e-learning environments by adapting content to individual learning styles. Eye-tracking provides insights into how learners interact with educational materials by analyzing gaze behavior, including fixation durations, saccades, and visual attention patterns.

#### 5.3.1. Identification of Learning Styles

The integration of eye-tracking technology can distinguish between visual and verbal learning styles by analyzing attention data, especially when content is presented in both text and graphics [69,70].

Nugrahaningsih et al. [70] conducted an eye-tracking study with 90 participants to distinguish between visual and verbal learning styles. They analyzed gaze data, specifically focusing on some features, including the percentage of fixation duration, percentage of fixations, etc. Participants viewed learning materials in a two-column layout, with graphics on the left and text on the right. The authors suggest a significant association between gaze behavior and learning styles. Eye-tracking technology may offer insights into cognitive processes related to aspects of the Felder-Silverman Learning Style Model (FSLSM), such as visual and verbal preferences.

Luo [71] identified four groups of learning styles in FSLSM. The author uses a Tobii eye tracker to collect gaze path and fixation duration. In addition, other behavioral patterns were selected to evaluate the accuracy of the proposed eye-tracking-based integration tool for identification compared with traditional self-report methods. The findings indicate that eye-tracking can identify different learning styles with accuracies ranging from 63.50% to 84.67%. The eye-tracking technology can be combined with other technologies, such as brain–computer interfaces. A systematic literature review by Jamil et al. [15] evaluates how emerging technologies like brain–computer interfaces and eye-tracking could enhance remote-learning environments. This study suggests that analyzing students’ brainwaves and eye movements can improve their cognitive abilities in online learning.

#### 5.3.2. Behavioral Patterns and Cognitive Styles

It primarily investigates the relationship between reading strategies and cognitive styles, verifying and exploring eye-tracking indicators of reading styles and their relation to cognitive processing [72]. To explore the relationship between reading strategies and cognitive styles, Lu et al. [73] employed eye-tracking methodology. They collected eye movement data using an SMI REDm eye tracker at 60 Hz. Their analysis revealed low-to-moderate correlations between mean saccade length and regression frequency across the reading materials. Based on these findings, the researchers suggest that this approach could be effectively applied to the design of foreign-language learning materials.

Khan et al. [74] presented the development of EXECUTE, a system designed to capture and analyze student attention during remote learning sessions using webcam-based gaze tracking. Integrating gaze metrics with sophisticated machine learning models, the system demonstrated an accuracy exceeding 91% in classifying students’ attention levels. This research contributes significantly to the potential for early identification and diagnosis of ADHD among students.

In their recent study, Négyesi [75] investigated the potential for automatically detecting visual and verbal learning styles in an adaptive e-learning system. They used the eye-tracking software GazeRecorder to record students’ interactions with learning materials. Subsequent analysis of the recorded eye-movement data revealed significant differences in gaze patterns between visual and verbal learners.

#### 5.3.3. Multimedia Learning and Information Processing

Eye-tracking technology is crucial for understanding how learners process multimedia content [76]. It enables the analysis of the effects of design features, presentation speeds, and visual cues on visual attention in multimedia learning contexts [77,78]. Additionally, it facilitates the study and enhancement of multimedia learning by examining learners’ visual attention and engagement with diverse types of educational materials [79].

To understand how label size and color affect multimedia learning, Hu and Zhang [78] employed eye-tracking technology. Using an Eyelink 1000Plus eye tracker, they analyzed learners’ visual attention patterns. Their findings contribute to our understanding of how visual cues in multimedia guide attention and enhance learning. Another work has demonstrated the benefits of using hands for learning and exploring potential explanations for these effects. Park et al. [79] provide a comprehensive review of previous studies on pointing and tracing in learning. Their eye movements were tracked to measure their attention and engagement during the learning operation. In the experimental step, the students were divided into three groups: one used pointing gestures, another used tracing gestures, and the third used no hand gestures. The results showed that the pointing group benefits from increased visual attention and cognitive activity.

#### 5.3.4. Course Recommendation

In e-learning, recommendation systems are pivotal for enhancing the learning experience by providing personalized guidance and support to individual learners. These systems can offer diverse recommendations, encompassing specific items, courses, and resources, contingent on the e-learning application’s objectives and requirements [80]. Integrating gaze data with deep learning models can significantly improve the performance of personalized online course recommendations by addressing challenges such as the cold-start problem and insufficient data. For instance, Chen et al. [81] demonstrated the efficacy of this approach by using eye tracking to capture users’ cognitive styles via heat maps and fixation-point trajectories. This approach enabled the system to deliver more tailored recommendations. The results highlighted that incorporating insights into cognitive style into recommendation systems fosters a more personalized and practical learning experience. Only one study within this category was identified in the literature. All the works cited previously are presented in Table 6.

### 5.4. Eye-Tracking Applications for Managing the Learning Content

The design and content type in e-learning environments significantly influence how learners interact with and absorb information [83]. Eye-tracking data reveal students’ natural gaze patterns, providing valuable insights into their attentional focus and engagement with content.

By analyzing these data, designers can optimize course layouts by strategically positioning critical information to enhance engagement and comprehension [84]. This approach ensures that e-learning environments are visually appealing and pedagogically effective, aligning content design with learners’ cognitive processes.

#### 5.4.1. Assessing the Impact of Different Design Elements

Assessing and refining design elements, e-learning systems can be optimized to foster better engagement, reduce cognitive load, and improve learning outcomes [85]. Using eye-tracking to assess design elements, e-learning developers can create visually engaging and pedagogically effective content, which improves learning experiences.

The influence of multimedia learning and visual design on student learning was examined using eye tracking in the study by Altan and Cagiltay [86]. Revised textbook content was evaluated for its impact on student achievement and learning behaviors. The resulting findings offer guidelines for developing student-centered science textbooks and multimedia resources, providing theoretical and practical implications for enhancing educational materials.

Mariñas et al. [87] conducted a study with 20 non-native English students in a synchronous online learning environment to investigate the impact of keyword color on reading comprehension and learning outcomes. Utilizing eye-tracking technology, they analyzed student gaze patterns while reading text with color-coded keywords. They found that red-colored keywords were most effective without time pressure, whereas they exhibited greater effectiveness under time constraints.

#### 5.4.2. Reducing the Cognitive Load

Reducing cognitive load can facilitate learning by optimizing the presentation of information and instructional design [88]. Eye-tracking studies show that aligning text and visuals can reduce cognitive load and improve information integration [89].

Dirkx et al. [90] evaluated whether instructional design guidelines based on CLT and CTML could improve the effectiveness of computer-based tests. Thirty-three students participated in an arithmetic exam, comparing their performance on two test items: the original and the redesigned item, in accordance with CTML principles.

Eye-tracking technology was employed by Sáiz-Manzanares et al. [91] to explore the rapport between cognitive load and learning outcomes. The researchers identified distinct cognitive-load clusters by comparing eye-tracking data across relevant and non-relevant areas of interest (AOIs). This work underscores the importance of further applied research to understand how cognitive load monitoring can personalize learning and enhance educational outcomes.

#### 5.4.3. Analyzing Student Interaction

Student interactions in e-learning are a crucial factor in determining the effectiveness of online courses. Active engagement with learning materials and peer interaction significantly enhances the learning experience by fostering a deeper understanding of the subject matter [92]. Eye-tracking provides objective, real-time data on how learners engage with various aspects of digital content, offering a window into their cognitive and behavioral processes during online learning [6].

Liu et al. [93] evaluated and enhanced digital learning content using eye-tracking technology to analyze eye movement behaviors and propose new metrics. They recorded eye movements using Tobii technology to assess three eye-movement metrics: fixation-time percentage, mean fixation duration, and spatial diversity of fixations. Participants evaluated the content and provided feedback on their level of interest. The findings revealed that the percentage of fixation time strongly indicates user interest, and that incorporating relevant semantic content positively influences engagement. This research offers guidelines for designing attractive digital learning materials.

Incorporating real-time gaze tracking into primary education presents exciting opportunities and significant challenges for enhancing teacher–student interactions. Educators can gain valuable insights into cognitive processes and classroom engagement dynamics by analyzing students’ eye movements, thereby improving learning outcomes [94].

Table 7 presents the works related to the application of eye-tracking in content learning.

### 5.5. Eye-Tracking Application for Learners’ Assessment

Effective teaching hinges on a teacher’s ability to accurately gauge two key aspects of their students: cognitive level and motivational–affective characteristics. The cognitive level encompasses a student’s prior knowledge of the subject matter, prerequisite skills, and current understanding of the taught concepts [41,97].

Motivational-affective characteristics refer to students’ intrinsic motivation to learn, emotional responses to the material (e.g., interest, frustration, anxiety), and overall attitude toward learning. Accurately assessing these factors allows teachers to understand where each student stands in their learning journey.

#### 5.5.1. Assess Reading Comprehension

Building on prior work in reading comprehension assessment, Mézière et al. [98] investigated eye tracking as an educational assessment tool. Comparing eye-tracking data with results from traditional reading comprehension tests, they demonstrated that eye-tracking measures offer more precise insights into comprehension than simply measuring reading speed.

The study of De-la-Peña [99] examined the application of eye tracking as a complementary tool for evaluating and improving implicit reading comprehension in young students. The research explored the relationships between these factors and implicit reading comprehension by analyzing eye-tracking metrics in relation to other factors, such as vocabulary, rapid automatized naming, and processing speed. The results suggest that eye tracking is a strong predictor of reading comprehension, providing evidence for its potential to optimize the assessment and development of reading skills in educational settings.

Other research focused on teachers to enhance the teaching process. For example, Kosel et al. [100] conducted a study investigating the cognitive processes underlying the professional task of student assessment as teachers perform it. Examining differences in eye-movement patterns (scanpaths), they found that expert teachers exhibit more complex scanpaths, frequently revisit all students, and distribute their attention evenly, leading to higher judgment accuracy than that of novice teachers.

#### 5.5.2. Enhance Teaching and Learning Outcomes

Eye-tracking technology has been recognized as a powerful tool to enhance learning and teaching outcomes by providing insights into learners’ cognitive processes, behaviors, and interactions [35,101,102]. IEyeGASE, an intelligent eye-gaze-based assessment tool developed by Ramachandra and Joseph [103], measures a range of learner behaviors during online assessments. These include wavering, confidence level, task engagement, completion speed, knowledge acquisition, and inattentional blindness. The research suggests eye tracking can provide valuable insights into learner performance and inform instructional practices.

Eye-tracking technology offers valuable insights into the learning and cognitive abilities of students with significant learning difficulties, as explored in Gill and Younie’s study [104]. This study can help teachers overcome challenges in evaluating the cognitive abilities of students who may struggle to communicate effectively. Combining heat maps, parent questionnaires, and classroom observations, this research provides more robust, independent data to inform teachers’ judgments and guide special-needs schools in investing in eye-tracking equipment to improve assessments.

Inter-subject correlation (ISC) of eye movements can be a reliable tool for assessing attention during online video learning, irrespective of learning styles, according to the research work of Su et al. [105]. This research explored the link between ISC and attention states, and the authors demonstrate that eye tracking can be used to deepen understanding of student engagement and attention in online learning environments, thereby informing the development of more effective and personalized teaching strategies. The findings suggest that ISC can be a reliable tool for assessing attention in video learning, regardless of individual learning styles.

#### 5.5.3. Understanding Student Performance

A thorough understanding of student performance is critical for optimizing e-learning environments and ensuring effective learning outcomes [106,107]. Understanding student performance through eye tracking enables educators and instructional designers to optimize teaching strategies and e-learning environments [53].

Kumar et al. [108] state that incorporating eye-gaze behavior can significantly enhance e-learning assessments. This approach goes beyond traditional scoring to offer a more comprehensive understanding of student performance. Classifying students into different reading pattern categories enables researchers to analyze the influence of these patterns on comprehension and question-answering abilities, leading to a categorization of students as ‘Novice,’ ‘Expert,’ or ‘Partial Knowledge. ’

In their 2024 study, Porras et al. [109] employed eye-tracking technology to collect data during arithmetic tasks, aiming to assess its effectiveness in capturing relevant eye-movement information in younger populations. This research enabled analysis of the relationship between eye-movement patterns and task performance, identifying specific visual-tracking patterns associated with distinct components of arithmetic calculations. The authors suggest that future research should expand the scope of eye-tracking applications to encompass a broader exploration of various aspects of mathematical cognition across diverse student populations.

#### 5.5.4. Cognitive Assessment

Cognitive assessment in e-learning refers to evaluating learners’ mental processes, such as thinking, reasoning, creativity, and problem-solving, within digital learning environments [110]. Eye-tracking metrics provide valuable data to evaluate learners’ cognitive and comprehension levels during complex tasks.

Standardized protocols are crucial in eye-tracking research, particularly when studying preverbal children, as highlighted in the work of Lenehan et al. [111]. This research used eye tracking to assess gaze behavior and identify potential atypical learning and behavioral patterns, underscoring the need for clear definitions of eye-tracking measures, calibration procedures, and detailed methodologies to ensure reliable and comparable results.

Juantorena et al. [112] developed and evaluated a new remote webcam-based eye-tracking prototype. This prototype addresses the challenges posed by lower camera quality and noisy environments, thereby making web-based experiments more practical. The work also introduces improvements to existing eye-tracking systems, such as eliminating the need for constant mouse interactions to optimize their use in cognitive and clinical tasks.

Table 8 presents works focusing on eye-tracking applications for learners’ assessment.

## 6. Findings, Discussion, and Limitations

Through this scoping review, we can deduce that eye-tracking studies on e-learning address a wide range of objectives, ultimately aiming to enhance the effectiveness and personalization of e-learning environments. Furthermore, eye-tracking studies on e-learning are developed for different aims, which often include:

- Analyzing student behavior: It offers a powerful tool for understanding learners’ behavior and improving the learning experience. Educators can leverage this technology to generate more engaging, personalized, and effective learning environments for all learners.
- Investigating cognitive processes: Researchers can gain deep insights into how learners process information and solve problems by analyzing eye movement patterns.
- Identifying learning styles: Research in this domain often centers on identifying patterns in ocular movements that correlate with specific learning preferences, such as visual and verbal learning styles.
- Improving content design: Eye-tracking studies analyze user interactions with e-learning interfaces, providing insights to optimize navigation, layout, and accessibility of learning platforms.
- Enhancing student assessment: It improves the accuracy and effectiveness of assessment across a wide range of domains and provides more objective, sensitive, and informative data on cognitive processes and behaviors.

A significant proportion of the reviewed studies investigated various aspects of visual attention, including fixation count, saccade rate, pupil dilation, and other relevant metrics. These metrics reveal cognitive processes during learning, thereby highlighting the crucial relationship between eye-movement patterns and the manifestation of visual attention in individuals [57].

These metrics are extracted from a variety of commercial and open-source tools. Web-based systems are generally more affordable and accessible than dedicated eye-tracking equipment. Meanwhile, web-based eye-tracking studies face a significant limitation, the constrained selection of gaze estimation algorithms. Using only a camera necessitates reliance on appearance-based methods, which often have lower resolution and frame rates, leading to less precise gaze data. Fixation analysis reveals crucial insights into learners’ interactions with various e-learning content formats. Studies demonstrate that fixation durations differ significantly based on the content type [86,87,93]. For instance, learners may exhibit longer fixation durations when interacting with dynamic animations compared to static infographics. However, this observation remains a hypothesis rather than a generalized finding, as it does not account for variations in task demands, sampling rates, or fixation algorithms across studies. Future research employing meta-analytic methods is needed to control for these variables and to confirm whether the increased visual complexity of dynamic content consistently leads to longer cognitive processing times.

More recent work integrates AOI-based analysis and scanpath modeling to understand interaction strategies better [91,115]. The most recent trend incorporates machine learning techniques to transform gaze features into predictive indicators of engagement, performance, and learner profiling.

These findings emphasize the importance of carefully balancing dynamic and static elements in e-learning designs. This approach ensures learner engagement while minimizing cognitive overload [90,95]. For example, subtle motion or gradual transitions can effectively guide attention and enhance learning without overwhelming the user. Several studies have proved that eye-tracking technology can effectively monitor students’ attention and engagement during online and remote learning sessions. This provides valuable insights to improve teaching strategies and facilitate early interventions for attention-related issues [46,47,49].

Wearable eye-tracking technology offers a valuable opportunity to assess and enhance classroom management techniques. Analyzing instructors’ visual attention patterns and interactions with classroom technology provides meaningful feedback for professional development [9,57]. However, integrating eye-tracking technology into classrooms poses challenges. These include device limitations and the need for accurate monitoring systems. Developing wearable and unobtrusive eye-tracking systems is crucial for practical implementation in educational settings [94]. Additionally, analyzing gaze data requires sophisticated models and techniques to accurately interpret student attention and engagement [79].

Some related studies aim to categorize and classify user states, including attention levels, emotional engagement, and cognitive load. To achieve this, these studies utilize machine learning techniques to analyze multivariate ocular feature sets, including fixation duration, saccade amplitude, pupil dilation, and gaze trajectory, to extract meaningful patterns from eye-tracking data [36,46,52].

Other studies have shown that eye-tracking technology can analyze the behavior of learners and teachers in educational settings. Keskin et al. [116] demonstrated its use with learners and teachers as participants, proving its applicability to student and teacher data. To study teachers’ behaviors, Daumiller et al. (2025) used eye-tracking technology to examine teachers’ cognitive processes and motivations related to their professional development.

To respond to the questions posed in the introduction, for the first one, we can say that eye-tracking technology enhances personalized learning experiences in several key ways:

- Real-Time Adaptation: Eye-tracking technology enables the immediate analysis of student attention and cognitive processes, paving the way for highly personalized learning experiences. Identifying gaze patterns associated with confusion or difficulty, the system can proactively initiate targeted interventions to support the learner in real time.
- Personalized Feedback and Support: Eye-tracking provides precise and individualized feedback by detecting areas where a student struggles. For instance, if a learner repeatedly fixates on a specific word or phrase, the system can offer contextual explanations or definitions to clarify the concept. This approach ensures that feedback is directly relevant to the learner’s needs, making it more effective than generalized responses.
- Adaptive Content Delivery: Learning platforms can dynamically adapt the presentation of content based on real-time eye-tracking data. If signs of inattention or disengagement are detected, the system can introduce interactive elements, gamified activities, or alternative formats to recapture interest. Additionally, it can prioritize the most relevant material based on the learner’s current focus and comprehension level, enhancing overall engagement and knowledge retention.

For the second question, several significant limitations affect the implementation of eye-tracking technology in educational settings:

- Technical and Implementation Challenges: Setting up and calibrating eye-tracking equipment can be time-consuming and require technical expertise. Portable and easy-to-use systems are needed for broader classroom implementation. Integrating eye trackers seamlessly with existing classroom technology and software can also pose an obstacle. Unfortunately, the costs of eye-tracking hardware and software can be prohibitive for many educational institutions.
- Data Management and Expertise Issues: Teachers need tools to quickly interpret data and translate it into actionable insights to improve teaching and learning. Developing robust, validated metrics for analyzing eye-tracking data in educational contexts remains an active area of research. A significant challenge in eye-tracking research is the lack of standardized protocols and methodologies. Different studies may use equipment, calibration procedures, data analysis techniques, and metrics to measure gaze behavior. This variability makes it complex to compare findings across studies and to synthesize the overall knowledge base.

Finally, for the last question, integrating eye-tracking data with other data sources, such as “learner’s data” or “learner’s performance data”, can create a more comprehensive and nuanced understanding of the learner experience in e-learning environments. This combination can improve the accuracy of predictive models for student success. For instance, a model might predict students’ risk of dropping a course based on their grades and engagement patterns, as revealed by eye tracking and learners’ data. Addressing these expertise issues is essential for the successful and ethical implementation of eye-tracking technology in education.

## 7. Conclusions

This scoping review highlights significant advancements in the application of eye-tracking methods in e-learning from 2020 to 2026. Eye-tracking offers a powerful lens into the learning process, providing a more nuanced and accurate measure of cognitive functions, such as reading comprehension, than traditional assessments. For instance, studies have shown that eye tracking can reveal subtle differences in how students process text, identifying areas of difficulty that traditional tests might miss.

Furthermore, the synergy between eye-tracking and advanced computational methods, such as machine learning and deep learning, has enhanced the potential of eye-tracking. These techniques enable automated analysis of complex eye-tracking data, allowing researchers to identify patterns and insights that would be impossible to discern manually. This integration paves the way for personalized learning experiences tailored to individual student needs and cognitive profiles. For example, machine learning algorithms can be trained to identify specific gaze patterns indicative of confusion or frustration, thereby enabling e-learning systems to dynamically adjust the difficulty or content of the presented material.

Synthesizing findings across various areas, this scoping review underscores the potential of eye-tracking technology to enhance our understanding of learner behavior, cognitive processes, learning styles, content effectiveness, and assessment strategies.

Furthermore, real-time eye-tracking use in e-learning environments holds immense promise for enhancing our understanding of student learning and informing more effective teaching practices. Also, real-time feedback on student engagement and attention enables instructors to adapt their teaching strategies, address learning gaps, and maximize student comprehension. For example, imagine an e-learning platform that can identify when a student is struggling with a particular concept and automatically provide additional resources or personalized support.

However, the widespread adoption of eye-tracking technology in education is not without challenges. Several key issues emerged from the analysis of previous works. These include the complexities of data interpretation, which require specialized training for educators to use the information gleaned from eye-tracking data effectively. Ethical considerations, particularly regarding student data privacy and the potential for technology misuse, must be carefully addressed. The cost of equipment and the technical expertise required for implementation are also significant barriers.

Although this scoping review identifies a clear convergence in the use of certain eye-tracking metrics for specific research goals, a definitive consensus on the most robust indicator for each educational outcome remains to be established.

Finally, the need for standardized protocols and methodologies across different studies is crucial for ensuring the reliability and comparability of research findings. Despite these challenges, the potential benefits of eye-tracking technology in education are substantial. Continued research and development are essential to address the identified challenges and maximize the positive impact of this technology on student learning outcomes.

Future research should focus on developing user-friendly tools for data analysis and interpretation, establishing clear ethical guidelines for data collection and usage, and exploring cost-effective solutions for implementing eye tracking in diverse educational settings. Resolving these challenges, we can harness the full potential of eye tracking to enhance learning experiences, making them more effective and personalized for all students.

## Figures and Tables

**Table 1:** Comparison of this work with previous literature reviews.

Review Reference	Main Focus	Environment	Articles Count	Differentiation
Alemdag and Cagiltay (2018) [12]	Cognitive processes (SOI model) and multimedia principles.	Laboratory/Desktop	52 articles (58 studies)	Extends their theoretical focus by addressing technical implementation in VR and webcam-based remote learning.
Wibirama et al. (2020) [13]	Comparison of approaches for detecting learning styles.	Multimedia learning	Brief review	Provides the missing comparative analysis they identified, focusing on the strengths/limitations of sensor-based AI integration.
Strohmaier et al. (2020) [14]	Mathematical thinking, mental representations, and subconscious processes.	Historical and Modern Math.	161 articles (1921–2018)	Addresses their call for more methodological rigor and critical data interpretation through standardized inclusion rationales.
Jamil et al. (2023) [15]	BCI and eye tracking for cognitive skills in online learning.	Remote Learning (Post-COVID)	44 articles	Addresses the real-time interaction gap they identified by focusing on the methodology for live feedback loops and automated analytics.
Da Silva et al. (2023) [16]	Perspective on neuroscientific paradigms in math education.	Naturalistic school settings	Perspective article	Transitions from their perspective on portable devices to a systematic evaluation of automated feedback loops in e-learning.
Andreou et al. (2024) [17]	Cognitive effort during text processing and comprehension.	Print vs. Digital media	13 articles	Complements their focus on reading strategies by providing a broader framework for real-time engagement in diverse e-learning tasks.
Chytry et al. (2025) [18]	PRISMA-based mapping of eye tracking in Education.	Web of Science/Multi-domain	Systematic Review	Updates their mapping with a specific focus on E-Learning 4.0 methodology and technical data-driven feedback.
The Present Work (2026)	Methodological Integration and Global Objectives	E-Learning (Remote/VR)	54 articles	Unique focus on categorizing global objectives, automated feedback, real-time analytics, and learner expertise modeling.

**Table 2:** Comparison of commercial and open-source eye-tracking tools.

Tool’s Name	Characteristics	Description	Limitation(s)
**Commercial Eye-tracking Tools**			
Tobii Pro	Typical spatial: 0.3–0.6°; Temporal resolution: 60–1200 Hz; Tracking technology: Infrared	A leading provider of eye-tracking solutions for research and industry. They offer a range of devices, from wearable glasses to desktop-mounted systems, as well as powerful analysis software [19].	It is costly, making them inaccessible for smaller institutions or individual researchers with limited budgets.
SMI (SensoMotoric Instruments)	Typical spatial: 0.39°–0.5°; Temporal resolution: Up to 1250 Hz; Tracking technology: Infrared	Another major player in the eye-tracking industry, SMI offers a variety of eye-tracking solutions for research, medical, and industrial applications [20].	SMI devices often require detailed calibration and controlled environments, which limit their flexibility for dynamic or real-world studies.
EyeTech	Typical spatial: 0.5°; Temporal resolution: 30 Hz–200 Hz; Tracking technology: Infrared	Known for its high-precision eye-tracking systems, EyeTech provides solutions for both research and clinical applications [21].	EyeTech devices are primarily focused on precision in research and clinical settings, which can limit their use in broader applications, such as real-world behavior tracking.
Eyelink	Typical spatial: 0.25–0.5°; Temporal resolution: 250–2000 Hz; Tracking technology: Infrared	Widely regarded as the gold standard for high-speed saccade and fixation analysis [22].	High cost and lack of portability make it unsuitable for large-scale use.
ISCAN	Typical spatial: 0.3–1°; Temporal resolution: 70–240 Hz; Tracking technology: Infrared	Provides versatile instrumentation for research, including head-mounted and remote tracking options [23].	Often requires proprietary software and complex integration for real-time automated feedback.
**Open-Source Eye-tracking Tools**			
WebGazer.js	Typical spatial: 1.4–4°; Temporal resolution: 30–50 Hz; Tracking technology: Visible light	A JavaScript library enabling eye tracking using standard webcams. It provides a valuable tool for researchers and developers willing to explore web-based eye-tracking applications without needing specialized hardware [24].	WebGazer.js’s accuracy is limited compared to infrared-based or commercial eye-tracking systems. It is suitable for general research but may not require high precision.
OpenSesame	Typical spatial: Depends on hardware; Temporal resolution: Depends on hardware; Tracking technology: Depends on hardware	A versatile open-source software platform for experimental psychology, including eye-tracking experiments [25].	OpenSesame relies on integration with external eye trackers. Its performance and usability depend on the hardware and plugins, which may not always be seamless.
Webcam-Based Eye-Tracking	Typical spatial: 1.5–5°; Temporal resolution: 1–50 Hz; Tracking technology: Visible light	Despite being calibration-free, webcam-based eye tracking, leveraging readily available devices, can effectively track user eye movements. This approach has demonstrated comparable accuracy to infrared-based eye tracking in predicting reading comprehension and detecting instances of mind wandering during reading tasks [26].	Factors such as lighting conditions, webcam quality, and participant positioning can affect tracking accuracy.

**Table 3:** Analysis of eye movement metrics.

Metric	Description
Fixation duration	The time the eye maintains a relatively stable gaze on a particular point or area of interest.
Fixation rate	The frequency of fixations per unit of time.
Average fixation duration	The average length of time that the eye fixates on a specific point of interest.
Saccade rate	The frequency of saccades within a specified time frame.
Average saccade length	Average distance the eye moves during a saccade.
Average saccade velocity	The speed of the eye during saccadic movements, usually measured in degrees per second.
Peak saccade velocity	Highest speed reached by the eye during a saccadic movement.
Heatmap	The regions where users looked most frequently or for the most prolonged duration, using a gradient of colors.
Pupil diameter	Refers to the size of the pupil, the black circular aperture in the center of the eye. It is quantified in millimeters (mm).
Pupil dilation	When that black circle becomes bigger to let in more light, like when we are in a dark room.
Scanpaths	The sequence and pattern of eye movements, including fixations and saccades.
Blink count	Number of blinks.

**Table 5:** Analysis of eye-tracking studies in e-learning according to cognitive processes.

Type of Affect	Author(s)	Objective(s)	Task(s)	Explored Feature(s)	Eye-Tracking Model	Participants
Reading and Comprehension Skills	[56]	Develop a predictive model of user interest levels by integrating gaze data with physiological signals.	Read 18 newspaper articles collected from the BBC News Database.	Fixation duration; saccade amplitude; saccade velocity; regression amplitude; regression velocity; saccade count; pupil diameter.	SMI (Remote)	13
	[5]	Detect task-unrelated thoughts and reading comprehension during online learning.	Participate in an online reading comprehension task.	Number of gaze points; number of fixations; fixation duration; heat map; saccade.	WebGazer.js (Webcam)	105
	[64]	Understand how students visually process and solve skill-based questions.	Answer a set of science questions.	Initial fixation time; first fixation duration; fixation count; mean fixation duration; total fixation duration; mean visit duration; summed visit duration.	Gaze Viewer (Software)	56
	[65]	Compare students’ reading patterns when presented with standard text and with text highlighting key terms.	Participate in reading sessions with key terms highlighted.	Fixation count; fixation duration; saccade.	WebGazer.js (Webcam)	80
	[57]	Explore the use of pre-questions as an active learning strategy to improve learning outcomes.	Read passages and answer pre-questions before or after reading.	Number of fixations, complete fixation time, and number of gaze transitions.	Eye Tribe (Remote)	24
Problem-Solving Skills	[59]	Develop a system to visualize students’ eye movements for educational research.	Solve a process while dragging tags into an equation.	Fixation count; fixation duration.	Tobii Pro X3-120 (Remote)	33
	[60]	Demonstrate how eye-tracking can offer teachers insights into cognition and teaching practices.	Record videos while students solve mathematical problems.	Fixation count; gaze heatmap.	Mangold Vision (Software)	19
	[58]	Investigate problem-solving behaviors when addressing geometry problems.	Solve geometry problems using eye-tracking technology.	Fixation duration; fixation count.	Tobii Studio (Remote)	8
	[66]	Analyze characteristics and trends in mathematics research.	Analyze existing theses (no direct student participation).	/	Tobii X2-60 (Remote)	12
Working Memory	[62]	Explore the effectiveness of eye-tracking training for children with learning difficulties.	Enroll in an eye-tracking training program or a traditional remediation class.	Fixation latency; saccade latency.	/	53
	[63]	Enhance automated detection and assessment of attention-related processes in learning environments.	Watch a recorded Zoom lecture on statistics.	Fixation duration; saccade.	SMI (Remote)	96

**Table 6:** Analysis of eye-tracking studies in e-learning according to learning styles.

Type of Affect	Author(s)	Objective(s)	Task(s)	Explored Feature(s)	Eye-Tracking Model	Participants
Identification of Learning Styles	[70]	Identify and investigate learning styles using an eye-tracking tool.	Look at the learning material in an e-learning course.	Fixation duration percentage; fixation count percentage; average fixation duration.	Eye Tribe ET-1000 (Remote)	90
	[71]	Explore the feasibility of integrating eye tracking to detect learning styles.	Look at and read the learning material and write a comment.	Gaze path; fixation duration.	Tobii (Remote)	30
	[82]	Inform the development of personalized and visually adaptive educational strategies.	Complete a series of cognitive tasks.	Gaze duration; fixation count; fixation duration; saccadic movements.	Tobii Pro Glasses 2 (Wearable)	20
Behavioral Patterns and Cognitive Styles	[73]	Investigate the relationship between reading strategies and cognitive styles.	Read a test and learning material.	Saccade.	SMI REDm (Remote)	24
	[74]	Utilize machine learning models to classify student attention levels.	Participate in remote teaching sessions.	Fixation; saccade.	WebGazer.js (Webcam)	25
	[75]	Automatically identify learning styles in an adaptive e-learning environment.	Interact with learning material.	Heat map; eye movement path.	GazeRecorder (Webcam)	255
Multimedia Learning and Information Processing	[78]	Examine how label size and color influence learners’ attention and retention.	Participate in a multimedia learning experiment and complete a retention test.	Fixation duration; glance count; fixation count.	Eyelink 1000 Plus (Remote)	61
	[79]	Investigate effects of touch-based actions (pointing and tracing) on learning.	Engage in a three-group experimental learning design.	Fixation duration; time to initial fixation (illustration); time to first fixation (text).	Tobii X2-60 Compact (Remote)	90
Course recommendation	[81]	Develop a model for recommending online courses tailored to individual learners.	Interact with an online course recommendation system.	Fixation duration; heat map; fixation point trajectory.	SMI RED (Remote)	20

**Table 7:** Analysis of eye-tracking studies in e-learning according to content types and design.

Type of Affect	Author(s)	Objective(s)	Task(s)	Explored Feature(s)	Eye-Tracking Model	Participants
Assessing the impact of different design elements	[86]	Explore how multimedia and visual design in science textbooks influences student learning.	Analyze student eye movements and attention patterns while interacting with the textbook.	Fixation duration; total fixation duration; fixations proportion.	Tobii X2-60 (Mobile)	80
	[87]	Investigate the impact of colored keywords on online learning.	Read materials containing keywords highlighted in different colors.	Fixation count; fixation duration; number of saccades; saccade duration; pupil diameter.	Tobii Pro Nano (Remote)	20
Reducing cognitive load	[90]	Measure cognitive load and eye movements to understand the effects of CTML-based design.	Complete a computer-based arithmetic exam.	Fixation duration.	SMI RED (Remote)	33
	[95]	Measure and analyze visual perception patterns to assess the effectiveness of training materials.	Participate in a training program with digital materials.	Fixation count.	Tobii Pro X3-120 (Remote)	15
	[91]	Identify clusters related to cognitive load in AOIs.	Engage with a self-regulated video in a virtual learning lab.	Fixation; saccade.	Tobii Pro (Remote)	42
	[96]	Examining the effect of visual presentation mode on cognitive load.	Perform remote simultaneous interpreting from English to Chinese in a technology-mediated environment.	Fixation duration; total time spent on an area of interest (AOI); number of fixations.	Tobii Pro X3-120 (Remote)	36
Analyzing student interaction	[93]	Analyze the relationship among eye movements, content, and evaluation scores.	View digital learning content (slide-deck-like works).	Percent of total fixation time; of total fixations; spatial dispersion of fixations.	Tobii (Remote)	65
	[94]	Increase interactivity, maintain engagement, and provide immediate feedback.	Fulfill all tasks presented by the game.	–	Hybrid (Remote + Wearable)	24

**Table 8:** Analysis of eye-tracking studies in e-learning according to learners’ assessment.

Type of Affect	Author(s)	Objective(s)	Task(s)	Explored Feature(s)	Eye-Tracking Model	Participants
Assess reading comprehension	[98]	Predict reading comprehension.	Read and answer questions about different text passages.	Fixation duration; saccade length; gaze duration.	EyeLink (Remote)	79
	[99]	Assess and improve implicit reading comprehension.	Complete implicit reading comprehension tests.	Fixation duration; fixation count; gaze duration.	Tobii Pro (Remote)	67
	[100]	Identify variations in eye-movement patterns among teachers with varying levels of expertise.	Watch a video depicting a typical learning scenario.	Fixation count; fixation duration; saccade.	SMI RED 500 (Remote)	35
	[113]	Evaluate the practicality and accuracy of webcam-based eye-tracking.	Read comic books.	Fixation.	GazeRecorder (Webcam)	22
Enhance teaching and learning	[103]	Create a tool to measure and analyze learner behavior during online assessments.	Participate in the online assessment system.	Fixation; saccade; blink count.	SMI (Remote)	15
	[104]	Enhance teacher evaluations for students with profound and multiple learning difficulties.	Participate in observation sessions in a familiar room.	Heat map.	Tobii PC EyeGo (Remote)	90
	[105]	Assess student attention in online learning.	Eye movements were tracked while students watched videos.	Pupil size; horizontal and vertical eye movements.	Video-based (Webcam)	29
Understanding student performance	[114]	Predict overall task performance by analyzing user interaction data.	Complete two masking tasks using Adobe Photoshop after watching a tutorial.	Fixation duration.	Tobii Pro (Remote)	53
	[108]	Explore how eye-tracking data offers additional insights beyond traditional scores.	Engage with assessment content.	Fixation count; fixation duration; saccade.	GazeRecorder (Webcam)	16
	[109]	Examine how eye movements relate to performance in mathematics.	Complete a computerized mental arithmetic task.	Fixation count; fixation duration.	Tobii Studio (Remote)	18
Cognitive assessment	[111]	Identify cognitive functions across different child cohorts.	Children observed while interacting with on-screen stimuli.	Fixation count; gaze points; time to first fixation; fixation sequences; first fixation duration; average fixation duration.	Tobii X2-60 (Remote)	16
	[112]	Develop and evaluate a remote webcam-based eye-tracking prototype.	Look away from a visual stimulus appearing on the screen.	Fixation; saccade.	Prototype (Webcam)	26

## Data Availability

No new data were created or analyzed in this study. Data sharing is not applicable to this article.

## Abbreviations

The following abbreviations are used in this manuscript:

ASD	Autism Spectrum Disorder
ADHD	Attention-Deficit/Hyperactivity Disorder
CLT	Cognitive Load Theory
CTML	Cognitive Theory of Multimedia Learning

## Appendix A. List of Included Studies and Rationale

This Appendix A provides the full list of primary studies analyzed in this scoping review, along with the specific justification for their inclusion.

**Table A1:** Rationale for including the selected studies.

Study	Year	Category	Objective	Reason for Inclusion
[33]	2021	Identifying Areas of Interest	Analyze teachers’ professional vision and compare novice and experienced teachers	Uses eye-tracking to analyze visual attention and professional vision in educational contexts
[34]	2022	Identifying Areas of Interest	Understand how students engage with live e-commerce content on Facebook Live	Investigates user attention and engagement using eye-tracking in digital media environments
[36]	2023	Classify User Behavior	Recognize unknown words during reading tasks	Uses gaze behavior to classify learner reading patterns
[53]	2020	Student Engagement	Measure learner attention during video-based learning activities	Uses eye-tracking to analyze engagement and attention in video-based learning environments
[46]	2023	Student Engagement	Analyze student behavior in virtual learning environments	Applies webcam-based eye-tracking to monitor learner interaction in online learning contexts
[52]	2024	Classify User Behavior	Classify user behaviors on websites	Uses gaze interaction patterns to model user behavior
[40]	2023	Predict Behaviors	Predict learning performance in embodied learning activities	Applies gaze-based indicators to model learning outcomes
[5]	2016	Improving Reading and Comprehension	Detect task-unrelated thoughts and analyze reading comprehension during online learning activities	Uses webcam-based eye-tracking to detect attention shifts and mind wandering during learning tasks
[60]	2021	Problem-Solving	Demonstrate how eye-tracking can provide teachers with insights into students’ cognitive processes during problem solving	Applies eye-tracking to support teaching practices and better understand students’ cognitive behavior
[70]	2021	Identification of Learning Styles	Identify and analyze students’ learning styles in an e-learning environment using eye-tracking	Investigates how visual attention patterns can reveal different learning strategies
[103]	2021	Enhance Teaching and Learning	Develop a tool to measure and analyze learner behavior during online assessments	Uses eye-tracking to monitor learner interaction and engagement during digital assessment activities
